# Infectious Disease: A Geographic Guide and Atlas of Human Infectious Diseases

**DOI:** 10.3201/eid1807.120604

**Published:** 2012-07

**Authors:** Bruno P. Petruccelli

**Affiliations:** Retired, Medical Corps, US Army

**Keywords:** infectious disease, geography, human infectious disease, United Nations, atlas, maps, geographic distribution

Infectious Disease: A Geographic Guide and Atlas of Human Infectious Diseases, 2 books recently published by Wiley-Blackwell, deliver to the global medicine bookshelf diagnostic adjuncts for expatriate clinicians and those who see immigrants or returning travelers, while also serving as pretravel references on regional disease risk and authoritative sources for anyone needing infectious diseases information. Mary Wilson, who contributed to the first book and wrote the foreword for the second, filled a similar need in 1991 with A World Guide to Infections. Now these new books remind us that even in the age of near–real-time, electronic references, a printed volume to hold in one’s hands can be an unmatched resource.

Infectious Disease: A Geographic Guide, edited by Eskild Petersen, Lin Chen, and Patricia Schlagenhauf, uses United Nations regions as an organizational basis, which achieves the objective of maintaining relevance with respect to by-country travel while reflecting the fact that pathogens do not recognize political borders. The regions are still country groupings, but the way this book cuts up the world integrates how transmission varies by topography, geoclimatic factors, and the fauna that include pertinent disease reservoirs and vectors. Well-written chapters also review background regional histories, evolving global disease patterns, and the impacts of migration, climate change, and public health interventions. Extensively published physicians who have experience in geographic medicine contributed to all of the book’s clinical content. Fifteen of the 22 region-specific chapters include authorship from within that region. Nicely organized tables dominate over paragraphs of text. Occasional inconsistencies occur in the use of a unique font that sets off headings and subheadings, but this is a relatively minor side effect of a first printing.

The sequence that reliably characterizes nearly all of the region chapters is by organ system, with diseases then addressed categorically by duration of symptoms, using a 4-week cutoff point. Additional sections cover adenopathy, fever without focal symptoms, eosinophilia with elevated IgE, antimicrobial drug resistance, vaccine-preventable diseases, and statistical summaries addressing economics, demographics, and mortality. However, some authors added sections with a syndromic, taxonomic, transmission-based, or incubation-based approach, and 4 of the chapters have more than a slight departure from the essential scheme. Nevertheless, the quality of the content is consistent and, in fact, is enhanced by the variations.

Overall, the Geographic Guide is an outstanding, quick reference for clinicians. The book provides a link between a patient’s history or travel itinerary on one hand and a differential diagnosis or guidance for preventive measures on the other.

From its title and external appearance, Atlas of Human Infectious Disease could be mistaken for a cytology or histology text. Once open, a geography book appears, and quickly enough, its pages reveal an essential visual almanac for anyone whose work confronts, or whose interests include, infectious diseases. This book shows the pictures we often seek but have difficulty finding: those that answer the question, “Where?” For a less common disease, where has it been reported? For a more common one, where is it not controlled? Part atlas and part disease manual, this work reflects an intensive effort by 120 contributors and reviewers, assuring the user of a broad, collective expertise. Oxford tropical disease researchers Heiman F.L. Wertheim and Peter Horby and ProMED mail co-founder John P. Woodall are the lead editors. The book provides taxonomic consistency with widely available sources such as the Control of Communicable Diseases Manual, and scientific articles specific to each topic were used extensively.

The first 40 of its 273 pages offer mapping of factors that influence disease transmission, clinical penetrance, and control. This portion could easily stand alone as a reference for a broad range of readers interested in any of numerous topics, from urbanization to climate to the global use of antibiotics and vaccines. The bulk of the book is a compendium of 2-page, clinical–epidemiologic summaries of human infections, each having the same left-page map and right-page text layout. A world map of equal area is the usual template for incidence and endemicity displays, whereas regional maps and insets are used as needed; however, no section or entry on geographically diverse, health care–associated bacterial infections is included. The entries do include selected opportunistic infections, but apart from agents of general public health importance such as bloodborne viruses, health care–associated infections are not given particular attention. On the other hand, doing so could easily have doubled the volume of the book.

Overlays on the maps are simple and usually contrast well with the core scheme to readily show relationships. For example, the outlining of vector distributions does not interfere with the use of solid colors to map disease occurrence. Where overlays would not work as well, the page includes one or more parallel maps, which may show inverse relationships such as immunization coverage versus disease incidence. The book indirectly begs for better surveillance by depicting large gray areas marked “No Data”; even the most developed countries often fail to escape this distinction. In fact, the type of passive reporting that supplied map data for some diseases leaves one wondering: is it “no data” or “no disease”?

Clever adjustments for reporting bias were made in some cases, though the nature of source data too often prohibits any valid attempt. Likewise, dependence on political borders to outline geographic distribution usually prohibits depiction of spatial density. When a disease has worldwide distribution, that fact is not usually evident in the map, which instead focuses on high-risk areas. This method is appropriate and reinforces the need to use the map and text pages together.

Purchasers are provided a code to download the book in various digital formats. Downloading is a fast and easy process, as is using the electronic version of the book itself. A single click on any topic or figure in the navigation pane takes the reader directly to the page desired. The resolution is excellent, and one can either scroll or page up and down through each entry. (An electronic book version of Infectious Disease: A Geographic Guide is also available from the publisher, but as a separate purchase from the print edition.)

The Atlas clearly sets a new standard as a geographic medicine reference and is certain to become an indispensable tool for epidemiologists and infectious diseases specialists. The editors hope it will also encourage the reporting of infectious diseases worldwide, which may well become its most important role.

